# How to Cope With Heavy Metal Ions: Cellular and Proteome-Level Stress Response to Divalent Copper and Nickel in *Halobacterium salinarum* R1 Planktonic and Biofilm Cells

**DOI:** 10.3389/fmicb.2019.03056

**Published:** 2020-01-17

**Authors:** Sabrina Völkel, Sascha Hein, Nathalie Benker, Felicitas Pfeifer, Christof Lenz, Gerald Losensky

**Affiliations:** ^1^Microbiology and Archaea, Department of Biology, Technische Universität Darmstadt, Darmstadt, Germany; ^2^Microbial Energy Conversion and Biotechnology, Department of Biology, Technische Universität Darmstadt, Darmstadt, Germany; ^3^Atmospheric Aerosol, Institute of Applied Geosciences, Technische Universität Darmstadt, Darmstadt, Germany; ^4^Bioanalytical Mass Spectrometry Group, Max Planck Institute for Biophysical Chemistry, Göttingen, Germany; ^5^Bioanalytics, Institute of Clinical Chemistry, University Medical Center Göttingen, Göttingen, Germany

**Keywords:** microbial communities, metal stress, extracellular polymeric substances, adhesion, scanning electron microscopy, proteome, SWATH-MS, label-free quantification

## Abstract

*Halobacterium salinarum* R1 is an extremely halophilic archaeon capable of adhesion and forming biofilms, allowing it to adjust to a range of growth conditions. We have recently shown that living in biofilms facilitates its survival under Cu^2+^ and Ni^2+^ stress, with specific rearrangements of the biofilm architecture observed following exposition. In this study, quantitative analyses were performed by SWATH mass spectrometry to determine the respective proteomes of planktonic and biofilm cells after exposition to Cu^2+^ and Ni^2+^.Quantitative data for 1180 proteins were obtained, corresponding to 46% of the predicted proteome. In planktonic cells, 234 of 1180 proteins showed significant abundance changes after metal ion treatment, of which 47% occurred in Cu^2+^ and Ni^2+^ treated samples. In biofilms, significant changes were detected for 52 proteins. Only three proteins changed under both conditions, suggesting metal-specific stress responses in biofilms. Deletion strains were generated to assess the potential role of selected target genes. Strongest effects were observed for ΔOE5245F and ΔOE2816F strains which exhibited increased and decreased biofilm mass after Ni^2+^ exposure, respectively. Moreover, EPS obviously plays a crucial role in *H. salinarum* metal ion resistance. Further efforts are required to elucidate the molecular basis and interplay of additional resistance mechanisms.

## Introduction

Biofilms, i.e., multicellular microbial communities embedded in a matrix of extracellular polymeric substance (EPS), are the predominant lifestyle adopted by microorganisms in nature (Flemming and Wuertz, 2019). While bacterial biofilms have been studied for decades, due to their clinical, industrial, and domestic relevance, knowledge about their archaeal equivalents is still limited. Numerous studies have demonstrated that biofilm formation is widespread among archaea and follows a similar life cycle, i.e., cell adhesion, accumulation, maturation, and disaggregation (Fröls, 2013; van Wolferen et al., 2018). Biofilms are dynamic systems and variable according to the environmental conditions (Chimileski et al., 2014; Zhang et al., 2019). Consequently, biofilm formation goes along with fundamental rearrangements at the cellular and molecular levels (Losensky et al., 2017).

Biofilms offer numerous advantages compared with the planktonic lifestyle. Living in biofilms favors microbial horizontal gene transfer by close proximity of the cells (Molin and Tolker-Nielsen, 2003). Cells in biofilms are prevented from releasing due to their immobilization, e.g., on solid surfaces. The biofilm matrix provides a hydrous gel-like environment and thereby protects from desiccation, but also hazardous environmental factors, such as antibiotics or toxic compounds like heavy metals (Ozturk and Aslim, 2010). The biofilm matrix acts as a diffusion barrier or binds diffusible compounds, facilitated by various functional groups on the cell surface or within the EPS (Wuertz et al., 2001; Martin et al., 2015).

Metal ions exert dual biological effects on microorganisms. On the one hand, they are indispensable for key physiological processes, e.g., as cofactors of enzymes or in redox processes. Metal ions, such as manganese, are involved in protein folding (Tannous and Kanaya, 2014) or even affect radiation resistance (Webb et al., 2013). Therefore, microbes have evolved specialized mechanisms for the uptake and storage of micronutrients, such as the iron chelator DpsA (Reindel et al., 2002; Zeth et al., 2004). On the other hand, the same metal ions can challenge and stress microbes when the concentrations become too high. They can either directly damage biomolecules or indirectly *via* catalysis of reactive oxygen species (ROS), that in turn have detrimental effects on cellular components (Igiri et al., 2018). For this reason, metal homeostasis is a crucial task for microorganisms. This is especially true for halophiles, since their habitats often serve as sinks for (heavy) metal ions, mainly as a consequence of water evaporation (Srivastava and Kowshik, 2013). These environments harbor (heavy) metal ion concentrations in a millimolar range depending on strongly variable water levels (Kumar et al., 2010; Pereira et al., 2013).

The most common mechanisms of (heavy) metal ion resistance include regulation of metal transport across membranes (influx/efflux), intra- or extracellular sequestration, and enzymatic detoxification (Voica et al., 2016). Examples for each of these mechanisms are known or predicted in the extremely halophilic archaeon *Halobacterium salinarum*. One mechanism is the active copper export *via* the P_1_-type Cu(II)-efflux ATPase YvgX as demonstrated in *H. salinarum* NCR-1 (Kaur et al., 2006). Moreover, uranium association to haloarchaeal cell surfaces was reported (Bader et al., 2017) as well as copper ion binding and storage, which is presumably mediated by copper-binding metallochaperones (Pang et al., 2013). Enzymatic detoxification, i.e., reduction or modification of the metal ions, such as methylation, were both proposed to contribute to arsenic resistance (Wang et al., 2004). The haloarchaeal iron homeostasis regulation is well established, comprising a network of four transcription factors of the DtxR family, i.e., Idr1, Idr2, SirR, and TroR (Martinez-Pastor et al., 2017). However, the presence of additional metal resistance factors is likely, and little is known regarding their interplay and regulation.

A systems analysis of the metal response performed with planktonic cells of *H. salinarum* NRC-1 demonstrates the potential of this species to differentiate between metals and to implement general or individual responses. Metal exposures lead to broad transcription adjustments, affecting 20% of genes (Kaur et al., 2006). We recently demonstrated higher survival rates of biofilm cells compared to planktonic cells upon heavy metal exposure of *H. salinarum* strain R1 (Völkel et al., 2018). Moreover, we observed metal ion-specific effects on biofilm architecture, i.e., increase or decrease of the cellular dense arrangement in mature biofilms after Ni^2+^ or Cu^2+^ treatment, respectively. Our transcriptional studies on several known and putative metal ion transporters suggested specific effects in consequence of the exposure (Völkel et al., 2018), but the underlying regulatory and cellular processes are unknown. Hence, a global approach to investigate the molecular basis for the biofilm metal ion resistance and rearrangements of the biofilm architecture is desirable.

The present study aimed to elucidate molecular processes contributing to metal ion resistance and altered biofilm morphologies in response to Ni^2+^ and Cu^2+^ treatment of *H. salinarum* R1. The EPS of treated and untreated biofilms was isolated, quantified, and its composition investigated with regard to proteins, carbohydrates, uronic acids as well as the metal ions under investigation. The proteomes of planktonic and biofilm cells exposed to nickel or copper ions were determined. Relative protein amounts were quantified using proteome profiling by SWATH mass spectrometry. Individual markers as well as biological processes were identified that showed significant and specific changes in response to metal ion treatment. A potential role of selected genes in metal response was further validated by complementary gene deletion studies. The generated deletion mutants were characterized with regard to their adhesion and biofilm formation capabilities by quantitative fluorescence-based assays or confocal laser scanning microscopy (CLSM). We also tested for sensitization to the respective metal ions by growth inhibition experiments.

## Materials and Methods

### Cultivation Conditions

*Halobacterium salinarum* R1 (ATCC 29341) was grown at 37°C in complex medium (4.3 M NaCl, 81 mM MgSO_4_, 27 mM KCl, 1.5% Oxoid peptone, 50 mM Tris/HCl pH 7.5). Planktonic cells were grown in cultures shaking at 180 rpm with a start optical density (OD_600_) of 0.02. At OD_600_ 0.3 metal ion solutions were added to specific growth-inhibiting but non-lethal concentrations, i.e., 5 mM Cu^2+^ and 15 mM Ni^2+^ (according to Völkel et al., 2018) and the cells were cultivated for another 24 h. Cells without metal ion treatment were cultivated as control. Adherent cells were statically grown in Petri dishes with a start OD_600_ 0.003. After 14 days of cultivation, the medium was supplemented with metal ion solutions to final concentrations of 5 mM Cu^2+^ and 40 mM Ni^2+^ (according to Völkel et al., 2018) and the biofilms were cultivated for another 24 h. At these concentrations, significant effects on biofilm architecture were observed, while the amount of dead cells was below 25% (Völkel et al., 2018). Untreated biofilms were used as control. For biofilm analysis, the supernatant was removed, dishes were washed three times with salt solution (4.3 M NaCl, 81 mM MgSO_4_, 27 mM KCl, 50 mM Tris/HCl pH 7.5), and biofilms were scraped from the dishes using a spatula.

### Scanning Electron Microscopy (SEM)

Biofilms were statically grown on carbon-coated gold grids, 400 mesh (Plano GmbH, Wetzlar, Germany) placed in Petri dishes with cultures inoculated with start OD_600_ 0.003. After 14 days of cultivation, the medium was supplemented with metal ion solutions and the biofilms were cultivated for another 24 h. Biofilms on grids were fixed with 2% paraformaldehyde (w/v) and 1% glutaraldehyde (w/v) over night at 4°C and contrasted with 2% uranyl acetate (pH 6, containing maleic acid) for 1 min. Samples were washed three times with distilled water, gently dried using a filter paper, and stored in a desiccator containing silica gel. To improve the image quality and resolution, the samples were sputter-coated with a 70 nm gold layer. Microscopic analysis was performed using a scanning electron microscope (FEI ESEM Quanta 200 FEG, Eindhoven, Netherlands) in combination with an energy dispersive X-ray silicon drift detector (X-Max Oxford Instruments, Abingdon, United Kingdom). The samples were investigated under high vacuum conditions with an acceleration voltage of 12.5 kV, spot size 4, and a working distance of 10 nm.

### Determination of Total Cell Counts

Planktonic cells and biofilms were exposed to copper and nickel ions for 24 h. Biofilm cells were scraped off the surface and the biofilm suspensions were vortexed for cell segregation. Planktonic and biofilm cell suspensions were diluted to approximately OD_600_ 0.5 before the total cell counts were determined by microscopy using a counting chamber (Neubauer-improved, depth 0.01 mm) and a Zeiss Axioskop 2. The cell counts are based on at least 60 counted squares (0.04 mm^2^ area) from two independently inoculated cultures. The significances (*p*-values) of changes in the amount of total cells in metal ion treated cells compared to untreated cells after 24 h were assessed by an unpaired, two-tailed *t*-test.

### Extraction of Extracellular Polymeric Substances (EPSs)

For EPS isolation, biofilms were scraped off the surface and 20 mL basal salt solution was added. Since the use of chemical extraction reagents caused damage of the *H. salinarum* cells, a physical method using ultrasound was used to isolate EPS. The biofilm suspensions were transferred into 50 mL polypropylene centrifuge tubes and treated by sonication in an ultrasonic water bath for 10 min. The water was cooled with ice, to avoid excessive heating. After sonication, the samples were stirred for 2 h at 4°C. Using a PMA-qPCR assay previously adapted to high salt conditions (Völkel et al., 2018), cell damage due to the isolation method was excluded. Each sample was divided into two parts; one part was centrifuged at 20,000 × *g* for 20 min (4°C) and the supernatant was filter-sterilized (0.45 μm pore size), the other part was directly used after stirring (biofilm part). The filtrate (EPS-fraction) and the biofilm part were dialyzed against deionized water at 4°C (molecular mass cut-off 3500 Da, 5× for a period of 2 days) and lyophilized. Prior to chemical analysis of EPS components, the dry masses were determined and the samples were suspended in buffer containing 100 mM Tris/HCl, pH 8.0, and 150 mM NaCl.

### Biochemical Analysis of EPS

Proteins, carbohydrates, and uronic acids were quantified in isolated EPS-fractions of the untreated, as well as the copper- and nickel ion treated biofilms. Protein concentrations were determined by a modified Lowry assay using bovine serum albumin as a standard (Frølund et al., 1996). For determination of carbohydrate concentrations, the phenol sulfuric acid method was applied using D-glucose as a standard (DuBois et al., 1956). The concentrations of uronic acid in EPS were determined by the colorimetric assay of Filisetti-Cozzi and Carpita (1991) using D-glucuronic acid as a standard. The significances of the respective assays were assessed by an unpaired, two-tailed *t*-test.

### Determination of Metal Concentration by Atomic Absorption Spectrometry (AAS)

The concentrations of Cu^2+^ and Ni^2+^ were determined in planktonic cultures and in biofilms after 24 h of metal exposure. Metal concentrations of planktonic cells were determined in cell cultures including the metal ion-supplemented medium as wells as in washed cell fractions. In biofilms, metal concentrations were determined in the metal supplemented medium, in washed and scraped off biofilms, as well as in extracted EPS- and cell fractions of biofilms. The samples were diluted with deionized water to reduce the amount of salts. Metal quantification was performed by atomic absorption spectrometry (AAS, contrAA^®^ 300, Analytik Jena AG) using standard solutions of 0–3 mg/L Cu^2+^ and Ni^2+^ dissolved in nitric acid. The quantification of the metal ions was based on three independent replicates, each consisting of three measurements.

### Sample Preparation for Mass Spectrometry

The sample preparation of planktonic and biofilm cells was performed in triplicate for each condition (untreated, Cu^2+^, or Ni^2+^ treated). Planktonic and biofilm cells were lyzed osmotically by the addition of 10 mM Tris/HCl (pH 7.5), and DNA in the cell lysate was hydrolyzed by treatment with 10 μg/mL DNase I. Protein concentrations were determined by a Bradford assay using bovine serum albumin as a standard. Equal protein concentrations were prepared by trichloracetic acid/acetone precipitation.

### Label-Free Proteome Profiling by SWATH Mass Spectrometry

50 μg protein per sample was loaded onto a 4–12% NuPAGE Novex Bis-Tris Minigels (Invitrogen) and run into the gel for 1.5 cm. Following Coomassie staining, protein areas were cut out, diced, and subjected to reduction with dithiothreitol, alkylation with iodoacetamide, and finally overnight digestion with trypsin. Tryptic peptides were extracted from the gel, the solution dried in a Speedvac, and kept at −20°C for further analysis (Atanassov and Urlaub, 2013). For generation of a peptide library, equal amount aliquots from each sample were pooled to a total amount of 80 μg, and separated into eight fractions using a reversed phase spin column (Pierce High pH Reversed-Phase Peptide Fractionation Kit, Thermo Fisher Scientific). All samples were spiked with a synthetic peptide standard used for retention time alignment (iRT Standard, Schlieren, Schweiz).

Protein digests were analyzed on a nanoflow chromatography system (Eksigent nanoLC425) hyphenated to a hybrid triple quadrupole-TOF mass spectrometer (TripleTOF 5600 +) equipped with a Nanospray III ion source (Ionspray Voltage 2400 V, Interface Heater Temperature 150°C, Sheath Gas Setting 12) and controlled by Analyst TF 1.7.1 software build 1163 (all AB Sciex). In brief, peptides were dissolved in loading buffer (2% acetonitrile, 0.1% formic acid in water) to a concentration of 0.3 μg/μL. For each analysis, 1.5 μg of digested protein was enriched on a precolumn (0.18 mm ID × 20 mm, Symmetry C18, 5 μm, Waters, Milford, MA, United States) and separated on an analytical RP-C18 column (0.075 mm ID × 250 mm, HSS T3, 1.8 μm, Waters) using a 90 min linear gradient of 5–35% acetonitrile/0.1% formic acid (v:v) at 300 nL min^–1^.

Qualitative LC/MS/MS analysis was performed using a Top25 data-dependent acquisition method with an MS survey scan of *m/z* 350–1250 accumulated for 350 ms at a resolution of 30,000 full-width at half-maximum (FWHM). MS/MS scans of *m/z* 180–1600 were accumulated for 100 ms at a resolution of 17,500 FWHM and a precursor isolation width of 0.7 FWHM, resulting in a total cycle time of 2.9 s. Precursors above a threshold MS intensity of 125 with charge states 2 +, 3 +, and 4 + were selected for MS/MS; the dynamic exclusion time was set to 30 s. MS/MS activation was achieved by CID using nitrogen as a collision gas and the manufacturer’s default rolling collision energy settings. Four technical replicates per reversed phase fraction were analyzed to construct a spectral library.

For quantitative SWATH analysis, MS/MS data were acquired using 65 variable size windows (Zhang et al., 2015) across the 400–1050 *m/z* range. Fragments were produced using rolling collision energy settings for charge state 2 +, and fragments acquired over an *m/z* range of 350–1400 for 40 ms per segment. Including a 100 ms survey scan this resulted in an overall cycle time of 2.75 s. Two replicate injections were acquired for each biological sample.

Protein identification was achieved using ProteinPilot Software version 5.0 build 4769 (AB Sciex) at “thorough” settings. A total of 166,604 MS/MS spectra from the combined qualitative analyses were searched against the UniProtKB *H. salinarum* R1 reference proteome (revision 12-2017, 2,570 entries) augmented with a set of 52 known common laboratory contaminants to identify 1,378 proteins at a false discovery rate (FDR) of 1%. Spectral library generation and SWATH peak extraction were achieved in PeakView Software version 2.1 build 11041 (AB Sciex) using the SWATH quantitation microApp version 2.0 build 2003. Following retention time correction using the iRT standard, peak areas were extracted using information from the MS/MS library at an FDR of 1% (Lambert et al., 2013). The resulting peak areas were then summed to peptide and finally protein area values, which were used for further statistical analysis.

Protein identification results were interrogated by Gene Ontology and KEGG enrichment using DAVID Functional Annotation Tool v6.8 (Huang da et al., 2009). The mass spectrometry proteomics data have been deposited to the ProteomeXchange Consortium via the PRIDE (Perez-Riverol et al., 2019) partner repository with the dataset identifier PXD015192.

### Statistical Analysis

All statistical analyses were performed using R version 3.4 (R Core Team, 2014). The normalized raw peaks were log_2_-transformed and the separability of the different groups was explored by principal component analysis (PCA). The different groups were compared pairwise in view of differentially produced proteins and the *p*-values were adjusted with the Benjamini–Hochberg method to control an FDR of 5% (Benjamini and Hochberg, 1995). The change in the protein production for each protein was quantified with the log_2_ fold change (log_2_FC) in comparison with the adjusted *p*-values. A significant change in protein abundance was determined with a log_2_FC > 1 or log_2_FC < −1 and an adj. *p*-value of 0.05.

### Generation of Gene Deletion Strains

Gene deletion strains were constructed using the pop-in/pop-out strategy (Koch and Oesterhelt, 2005). Approximately 500 bp upstream (US) and downstream (DS) regions of the gene of interest were amplified by PCR and the sequences were fused resulting in the lack of the gene of interest in between. The fragments were cloned into plasmid pMKK100 carrying the gene *bgaH* encoding a halophilic beta-galactosidase. The plasmid for deletion of OE2816F was constructed using the NEB Gibson Assembly Cloning Kit (NEB #E5510) according to the manufacturer’s protocol. Oligonucleotides used are listed in Supplementary Table S1. *H. salinarum* R1 was transformed with the deletion plasmids by the polyethylene glycol method (Dyall-Smith, 2008) and plated on agar media containing mevinolin (6 μg/mL) and X-gal (40 μg/mL) to allow blue-red screening. Blue colonies, indicating plasmid integration by hydrolysis of X-gal, were selected and regrown at least three times in liquid cultures without mevinolin to allow the pop-out event. Cultures were plated on agar media containing X-gal and red colonies were screened for the deletions of the genes of interest by PCR using US/DS flanking oligonucleotides (Supplementary Table S1). Deletions were verified by sequencing analyses of the respective PCR products.

### Growth Curves

Growth of *H. salinarum* R1 and deletion strains was investigated by measuring the optical density (OD_600_) starting with OD_600_ 0.02 for a period of 97 h.

### Adhesion Assay

Adhesion of *H. salinarum* R1 and deletion strains was investigated by a fluorescence-based adhesion assay. The respective *H. salinarum* R1 strains were cultivated in 24-well microtiter plates for 15 days at 37°C. Acridine orange (Merck KGaA) was added to a final concentration of 1 μg/mL to each cavity and incubated for 15 min in the dark to label the adherent cells. The wells were washed three times with salt solution to remove planktonic cells, and the fluorescence signals of adherent cells were measured using a PhosphorImager (FLA 5000, Fujifilm) and Image Reader FLA 5000 Series software. The fluorescence signals were quantified using the software Image Gauge V4.23. To determine the significance of changes in adhesion, an unpaired, two-tailed *t*-test was performed.

### Agar Diffusion Assay

The sensitivity of gene deletion strains to metal ions was investigated by agar diffusion assays. Complex medium agar (1.5% [w/v] agar) was covered with complex medium soft agar (1% [w/v] agar) inoculated with *H. salinarum* R1 or deletion mutants. After the soft agar solidified, filter papers soaked with metal ion solutions (1 M Cu^2+^; 2.5 M Ni^2+^) or deionized water (control) were placed on the agar surface and the plates were incubated for 8 days at 42°C. The sensitivity of strains to metal ions was determined by measuring the diameter of growth inhibition around the filter paper. The significances of changes in the inhibition radius were evaluated by an unpaired, two-tailed *t*-test.

### Confocal Laser Scanning Microscopy (CLSM)

The architecture of *H. salinarum* R1 and deletion strain biofilms exposed to metal ions was investigated by confocal microscopy. Biofilms were grown in small Petri dishes (35/10 mm, Sarstedt) on polyethylene terephthalate surfaces in complex medium for 14 days. Metal ion solutions were added to final concentrations of 5 mM Cu^2+^ and 40 mM Ni^2+^ and the biofilms were cultivated for another 24 h. Biofilms were stained using acridine orange (10 μg/mL final) to visualize cells and concanavalin A (ConA) Alexa Fluor 647^®^ conjugates (Life Technologies, 20 μg/mL final) for the visualization of glycosidic (α-mannopyranosyl and α-glucopyranosyl) residues in EPS. Staining was performed for 15 min in the dark followed by washing the biofilms three times with 2 mL salt solution to remove non-adherent cells and unbound stains. Microscopic analysis was performed using a confocal laser scanning microscope (TCS SP5 II, Leica Microsystems GmbH, Wetzlar, Germany) in combination with the Leica Application Suite software and images were processed with ImageJ software. The biofilm surface coverage was quantified using ImageJ. At least eight independent micrographs were included in the analyses for each strain and condition, respectively. The significance was assessed by an unpaired, two-tailed *t*-test.

## Results

### Influence of Metal Ions on the Biofilm Architecture and Cell Number in *H. salinarum* Biofilms

**FIGURE 1:**
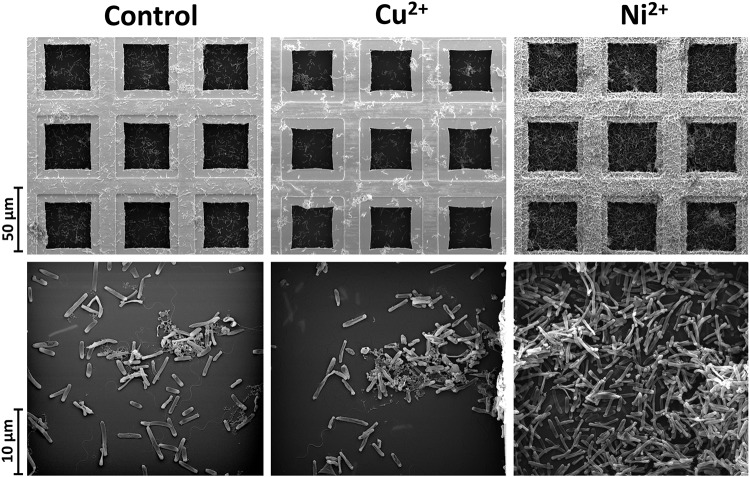
Scanning electron microscopy (SEM) of *H. salinarum* biofilms without metal treatment (control) and after 24 h treatment with 5 mM copper or 40 mM nickel ions.

**FIGURE 2:**
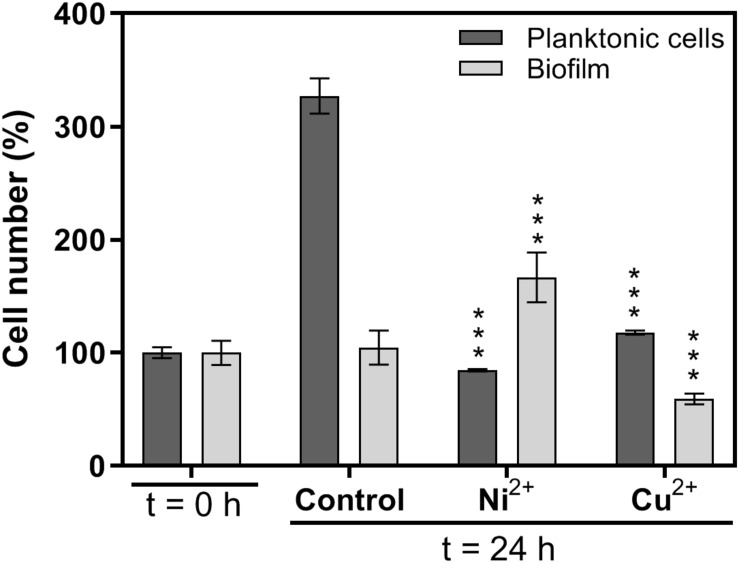
Effects of metal ion treatment on cell numbers in planktonic cell cultures and biofilms. Biofilms were scraped from the surface and dispersed. Cell numbers in cell suspensions were determined using a counting chamber. The numbers of planktonic cells (dark bars) and biofilm cells (light bars) were determined before (*t* = 0 h) and after (*t* = 24 h) 24 hours incubation in the absence (control) or presence of Ni^2+^ or Cu^2+^, respectively. Planktonic cells: 5 mM Cu^2+^, 15 mM Ni^2+^, biofilms: 5 mM Cu^2+^, 40 mM Ni^2+^. Planktonic cells: 100% $\hat{=}$ 2.6^∗^10^8^ cells/mL, biofilm cells: 100% $\hat{=}$ 8.0^∗^10^8^ cells/mL. The significance of the total cell number between metal-exposed cells and the control after 24 h was assessed by *t*-test (^∗∗∗^ extremely significant = *p* < 0.001).

The effects of copper and nickel ions on mature *H. salinarum* biofilms were investigated by scanning electron microscopy (SEM). Mature biofilms were supplemented with final concentrations of 5 mM Cu^2+^ or 40 mM Ni^2+^ and incubated for another 24 h. Untreated biofilms were used as control. SEM analysis of the control biofilms showed individual sessile cells spread over the grid and some small cell aggregates surrounded by an extracellular matrix (Figure 1). Treatment with copper ions resulted in detachment of single cells and the formation of larger and denser cell aggregates on the surface. In contrast, exposure to nickel ions resulted in significantly increased numbers of sessile cells in dense multilayers spread over the entire grid surface (Figure 1). Cell counting yielded constant cell numbers in control biofilms, whereas Cu^2+^ treatment resulted in a decrease to 60% and Ni^2+^ treatment in an increase to 170% of the cells, respectively (Figure 2). In planktonic cultures without metal ion exposure, the cell numbers increased to 330% after 24 h in comparison to biofilms. The planktonic cells were treated also with growth-inhibiting but non-lethal concentrations of 5 mM Cu^2+^ or 15 mM Ni^2+^, and treatment with Cu^2+^ resulted in a slightly increased cell number (118%), whereas the exposure of Ni^2+^ resulted in a decreased cell number (85%) (Figure 2).

### Effects of Metal Ions on Extracellular Polymeric Substances in Biofilms

**FIGURE 3:**
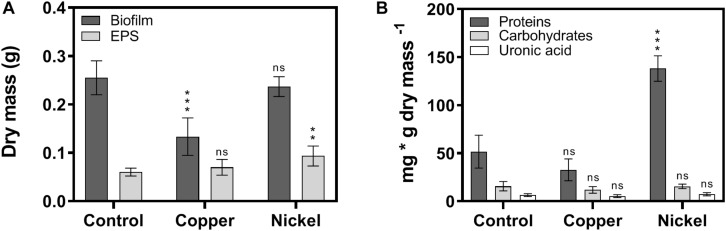
Quantitative investigations on biofilm and EPS composition after exposure to metal ions. **(A)** Dry mass of lyophilized biofilms and isolated EPS-fractions exposed to Cu^2+^ and Ni^2+^. **(B)** Quantifications of proteins, carbohydrates, and uronic acids in isolated EPS-fractions. The significance of the quantifications of metal ion-treated samples compared to the control was assessed by *t*-test (ns, not significant, ^∗∗^ highly significant = *p* < 0.01, ^∗∗∗^ extremely significant = *p* < 0.001).

The quantity and composition of EPS in biofilms was determined using isolated EPS either untreated or treated with metal ions to investigate potential effects of Cu^2+^ and Ni^2+^ on the composition of the biofilm matrix. Biofilm and isolated EPS-fractions were lyophilized and the dry mass was determined. In the case of biofilms, the dry mass of the control and after Ni^2+^ treatment was about 0.25 g, while the dry mass in Cu^2+^ treated biofilms was reduced to 0.13 g (Figure 3A). In EPS-fractions, the dry mass was 0.06 g in case of the control, 0.07 g in Cu^2+^ treated, and 0.09 g in Ni^2+^ treated biofilms. The resulting EPS to biofilm mass ratio was 22% in control biofilms, while the amount of EPS in Ni^2+^ and Cu^2+^ treated biofilms was significantly increased to 39 and 55%, respectively.

Investigation of the composition of isolated EPS showed that the untreated control biofilm mainly consisted of proteins amounting to 52 mg/g dry mass, followed by carbohydrates (16 mg/g dry mass) and uronic acids (6 mg/g dry mass) (Figure 3B). While the amounts of carbohydrates and uronic acids were similar in EPS of metal treated biofilms, the amount of proteins was decreased to 33 mg/g dry mass in Cu^2+^ treated and increased to 138 mg/g dry mass in Ni^2+^ treated biofilms (Figure 3B).

To determine the metal ion concentration in the cellular environment, quantitative measurements of Cu^2+^ and Ni^2+^ in planktonic cell cultures as well as in biofilms were performed. Planktonic cell cultures were treated with 15 mM Ni^2+^ or 5 mM Cu^2+^ for 24 h, and the metal concentrations were determined in liquid cultures (medium and cells) as well as in washed cells. In Cu^2+^ treated samples, the amount of Cu^2+^ was 4 mM in cell cultures and 30 μM in washed cells, whereas Ni^2+^ treatment resulted in 13.5 mM Ni^2+^ in cell cultures and 95 μM Ni^2+^ in washed cells (Table 1). In the case of biofilms, the metal ions were supplied as 40 mM Ni^2+^ or 5 mM Cu^2+^ for 24 h. The concentrations of the metal ions were quantified in the culture supernatant, in scraped off biofilms as wells as in isolated EPS- and cell fractions. In Cu^2+^ exposed samples, 4.6 mM Cu^2+^ was determined in culture supernatants, while in biofilm samples, only 12.6 μM Cu^2+^ was found, of which 8.2 μM was determined in EPS- and 0.9 μM in the cell fraction (Table 1). In the case of Ni^2+^ exposure, 32.7 mM Ni^2+^ was obtained in culture supernatants, 92 μM in biofilm samples, 76.7 μM in EPS-, and 10.2 μM in cell fractions (Table 1).

**TABLE 1:** Concentrations of copper and nickel ions in planktonic cell cultures and in biofilms.

	**Planktonic cells**		**Biofilms**			
	**Liquid culture (mM)**	**Washed cells (μM)**	**Supernatant (mM)**	**Washed biofilm (μM)**	**EPS (μM)**	**Cells (μM)**
Cu^2+^	4±0.26	30±3	4.6±0.6	12.6±5.5	8.2±0.5	0.94±0.2
Ni^2+^	13.5±1.3	95±3.4	32.7±3.6	92±10.9	76.7±2.6	10.2±0.7

### Proteome Profiling in Metal-Exposed Biofilms and Planktonic Cells

Quantitative proteome profiling was performed by label-free mass spectrometry to look for global adaptions to heavy metal ion stress on the proteome level. The generation of a project-specific spectral library resulted in a total of 1378 identified proteins at an FDR of 1%, corresponding to a proteome coverage of 54%. Analysis by Gene Ontology and KEGG enrichment determined these proteins to be largely cytosolic and belonging to a range of metabolic and biosynthetic pathways. While enrichment analysis is not representative at a GO and KEGG annotation rate of just below 40% of the detected proteins, it still allowed us to conclude that our data provides good coverage especially for cell metabolism (Supplementary Table S2).

The spectral library was then used to extract protein expression profiles from quantitative LC/MS/MS acquisitions employing SWATH (Sequential Window Acquisition of All Theoretical Precursors) mass spectrometry. Using this data-independent acquisition strategy, consistent quantitative profiling of 1180 proteins was achieved at an estimated FDR of 1% and at < 1% of missing values, corresponding to 46% of the predicted proteome. Following normalization, these proteome profiles were analyzed for significant changes under metal stress conditions.

**FIGURE 4:**
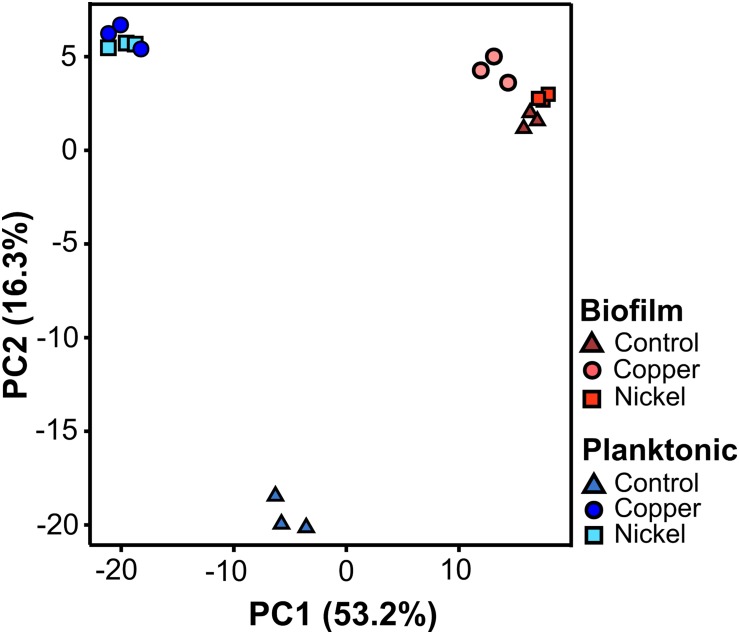
Principal component analysis of the quantitative proteome data sets of planktonic (blue) and biofilm (red) samples. For each physiological state, three independent biological replicates of untreated (triangles), copper (dots), and nickel (squares) treated samples were investigated. Planktonic cells were grown to an optical density of 0.3 and then treated with 5 mM Cu^2+^ and 15 mM Ni^2+^ for 24 h. Biofilms were grown for 14 days and then incubated with 5 mM Cu^2+^ and 40 mM Ni^2+^ for another 24 h.

A principal component analysis (PCA) was performed to evaluate the degree of variation between the treated and untreated samples, and also between planktonic and sessile (biofilm) cells. The PCA is based on the two largest components PC1 and PC2 combined accounting for 69.5% (Figure 4). Regarding planktonic cells, metal ion (Ni^2+^ or Cu^2+^) treatment led to a clear differentiation compared to the untreated controls, while the treated samples clustered closely among each other (Figure 4). The biofilm samples were distinct compared to the planktonic untreated as well as planktonic treated specimen. Considering only the biofilm samples, they all clustered relatively closely, independent of the treatment. However, the differently treated biofilm specimens were still distinguishable due to clustering of the biological replicates (Figure 4).

**FIGURE 5:**
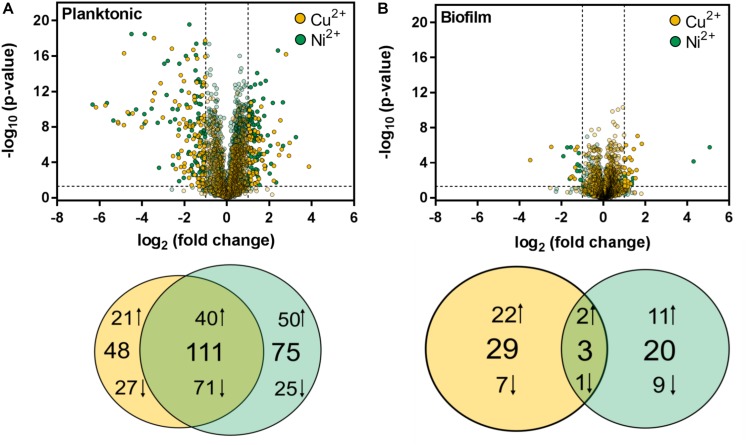
Statistical visualization of the protein abundances after copper and nickel treatment compared to the control in **(A)** planktonic and **(B)** biofilm samples. Proteins are ranked in a volcano plot according to their *p*-value and their relative abundance ratio between copper/nickel treated and control samples. Proteins with *p*-values < 0.05 and fold changes (FC) ≤ 0.5 and ≥ 2, respectively, are shown in Venn diagrams illustrating the number of equal and unique proteins after copper and nickel treatment. Arrows up indicate increased (FC > 2) and arrows down indicate decreased (FC < 0.5) protein abundances compared to the control.

For statistical analysis, the proteome data of metal ion treated samples were compared to the control for planktonic and biofilm specimens, respectively. Proteins with a fold change < 0.5 and > 2, respectively, and a significance level (adjusted *p*-value) < 0.05 was further regarded as proteins with significantly changed abundances. In the case of planktonic samples, a total of 234 proteins exhibited significant changes in metal ion treated samples compared to the control. Among these, 111 proteins were found in both copper and nickel ion treated samples, whereas 48 proteins occurred exclusively in Cu^2+^ treated and 75 proteins exclusively in Ni^2+^ treated samples (Figure 5). Metal ion exposure of planktonic cells mainly resulted in an increase of proteins belonging to the category “information storage and processing” with 41 and 30% in the case of Cu^2+^ and Ni^2+^ treatment, respectively. These include for example ribosomal subunits, diverse transcription regulators or the transcription initiation factor IIB, implying global transcriptional changes in response to metal ions. In comparison, the largest part of decreased proteins (46 and 42% in case of Cu^2+^ or Ni^2+^ treatment, respectively) consisted of proteins in cell metabolism dominated by a variety of ABC transporter proteins (Supplementary Table S3).

In biofilms, only 52 proteins exhibited significant changes in metaltreated samples compared to the control, with only three proteins overlapping between Cu^2+^ or Ni^2+^ (Figure 5) treatment. The largest fraction of the proteins, notably 29 in the case of copper and 20 in the case of nickel, were found exclusively, indicating metal-specific responses in biofilm cells. Cu^2+^-specific changes in both planktonic and biofilm cells are mainly based on an increase of transcription factors, whereas Ni^2+^-specific changes are based on proteins with unknown function (Supplementary Table S3).

### Construction and Characterization of Gene Deletion Strains

Seven gene deletion strains were constructed from *H. salinarum* R1 to investigate the effects of copper and nickel ions on planktonic cells and biofilms. Target genes were selected based on the protein abundances in metal ion treated samples compared to the control as determined in this study (Table 2). The uncharacterized protein B0R587 and the transcription regulator B0R8N0 showed high abundances in both, planktonic and biofilm, samples treated with copper or nickel ions. B0R9Z2 is part of an ATP-binding system and showed a high abundance in metal ion treated biofilm samples, and a low abundance in planktonic samples. Also, the predicted protease B0R859 showed low abundances in the planktonic samples, while it was highly abundant in Ni^2+^ treated biofilms. Metal-specific reactions were observed for an ATPase (B0R3Z0), showing high abundances in Cu^2+^ treated planktonic samples, and for a HMA domain protein (B0R3Z1) and an ABC transporter subunit (B0R9T5) with high abundances exclusively in planktonic samples exposed to Ni^2+^. Fold changes and functional categories of the selected proteins and the encoding genes are listed in Table 2.

**TABLE 2:** Target genes selected for gene deletion studies based on our proteomic data.

**Gene**	**Protein**	**Category**	**Function description**	**Fold change in abundance compared to untreated sample**			
				**Biofilm Cu^2+^**	**Biofilm Ni^2+^**	**Planktonic Cu^2+^**	**Planktonic Ni^2+^**
OE2816F	B0R587 uncharacterized protein	Poorly characterized	Function unknown	2.03	33.65	2.36	3.81
OE4612F *hly*	B0R859 serine protease halolysin	Cellular processes and signaling	Posttranslational modification; protein turnover; chaperones	0.99	19.65	0.06	0.07
OE5245F	B0R9Z2 ABC-type transport system ATP-binding protein	Cellular processes and signaling	Defense mechanisms	2.09	2.67	0.22	0.17
OE5146R *znuC*	B0R9T5 ABC-type transport system ATP-binding protein	Metabolism	Inorganic ion transport and metabolism	0.78	0.58	1.16	2.09
OE2042F *copA*	B0R3Z0 P-type transport ATPase	Metabolism	Inorganic ion transport and metabolism	0.86	0.85	4.64	0.71
OE2044F	B0R3Z1 HMA domain protein	Metabolism	Inorganic ion transport and metabolism	1.71	1.50	1.11	4.15
OE6177F	B0R8N0 PadR family transcription regulator	Information storage and processing	Transcription	2.27	2.17	3.44	2.43

**FIGURE 6:**
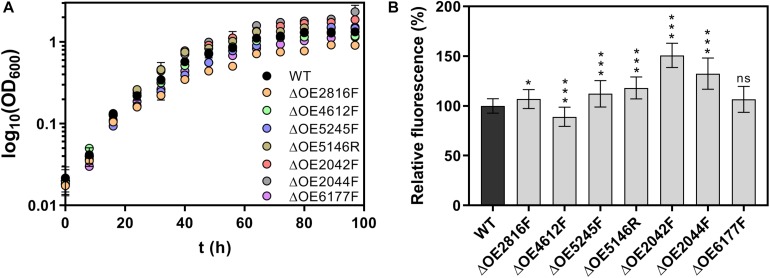
Characterization of *H. salinarum* R1 wild type (WT) and gene deletion strains. **(A)** Growth curves of *H. salinarum* strains with a start optical density (OD_600_) of 0.02 cultivated over periods of 97 h in complex medium. **(B)** Fluorescence-based adhesion assays of *H. salinarum* strain after 15 days of cultivation in complex medium. The diagram shows the relative fluorescence based on adherent cells of the deletion strains relatively compared to the wild type (set to 100%). The significance of the adhesion in gene deletion strains compared to the wild type was determined by *t*-test (ns, not significant, ^∗^ significant = *p* < 0.05, ^∗∗∗^ extremely significant = *p* < 0.001).

**FIGURE 7:**
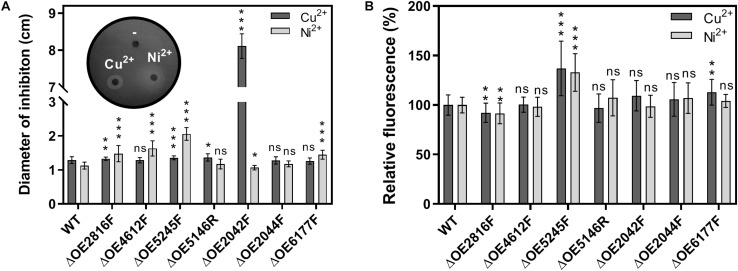
Characterization of *H. salinarum* R1 wild type (WT) and deletions strains exposed to metal ions. **(A)** Agar diffusion assays testing the metal sensitivity by measuring the zones of growth inhibition surrounding Cu^2+^ or Ni^2+^ soaked filter papers. **(B)** Fluorescence-based adhesion assays. Biofilms were grown in complex medium for 14 days and sessile cells were treated with Cu^2+^ or Ni^2+^ for 24 h. The diagram shows the relative fluorescence based on adherent cells of the deletion strains relatively compared to the wild type (set to 100%). The significance of the quantifications in gene deletion strains compared to the wild type strain was assessed by *t*-test (ns, not significant, ^∗^ significant = *p* < 0.05, ^∗∗^ highly significant = *p* < 0.01, ^∗∗∗^ extremely significant = *p* < 0.001).

Results of the characterization of the deletion mutants including growth curves, adhesion assays and metal sensitivity assays are shown in Figures 6, 7. Starting with an optical density at 600 nm (OD_600_) of 0.02, *H. salinarum* R1 wild type grew to the stationary phase with a final OD_600_ 1.3 in a period of 97 h. Most deletions strains tested showed a similar growth behavior, whereas the mutant strains ΔOE2044F and ΔOE2042F reached higher final optical densities of 2.3 and 1.9, respectively. In contrast, ΔOE2816F showed a slower growth compared to the wild type, reaching OD_600_ 0.9 after 97 h (Figure 6A).

Surface adhesion of deletions mutant strains compared to the wild type strain R1 was investigated by a fluorescence-based adhesion assay after 15 days of cultivation. Surface adhesion was strongly increased to 151 and 132% in case of ΔOE2042F and ΔOE2044F, respectively, while the remaining deletion strains also showed significant effects on adhesion (with the exception of ΔOE6177F), but to a smaller extent compared to the wild type (Figure 6B).

Agar diffusion assays were performed to test whether the gene deletions affected metal sensitivity of the cells (Figure 7A). Application of Cu^2+^ resulted in well-defined inhibition zones with diameters of 1.3 cm in the wild type strain. Most deletion strains tested showed similar effects and values. In contrast, ΔOE2042F displayed an extremely increased sensitivity to Cu^2+^ with inhibition zones of approximately 8 cm. Application of Ni^2+^ gave rise to inhibition zones of about 1.1 cm in the wild type. The deletion strains ΔOE2816F, ΔOE4612F, ΔOE5245F, and ΔOE6177F showed increased sensitivity to Ni^2+^, with ΔOE5245F showing the strongest effects, i.e., 2.0 cm inhibition zones.

Adhesion assays of the wild type and deletion strains were conducted after metal ion treatment, to test whether the deletions influence the adhesion in the presence of Cu^2+^ or Ni^2+^, respectively (Figure 7B). Altered adhesion upon metal exposure was only observed in case of ΔOE5245F. In this deletion variant, both, copper and nickel ions, resulted in approximately 30% stronger adhesion signals compared to the wild type.

**FIGURE 8:**
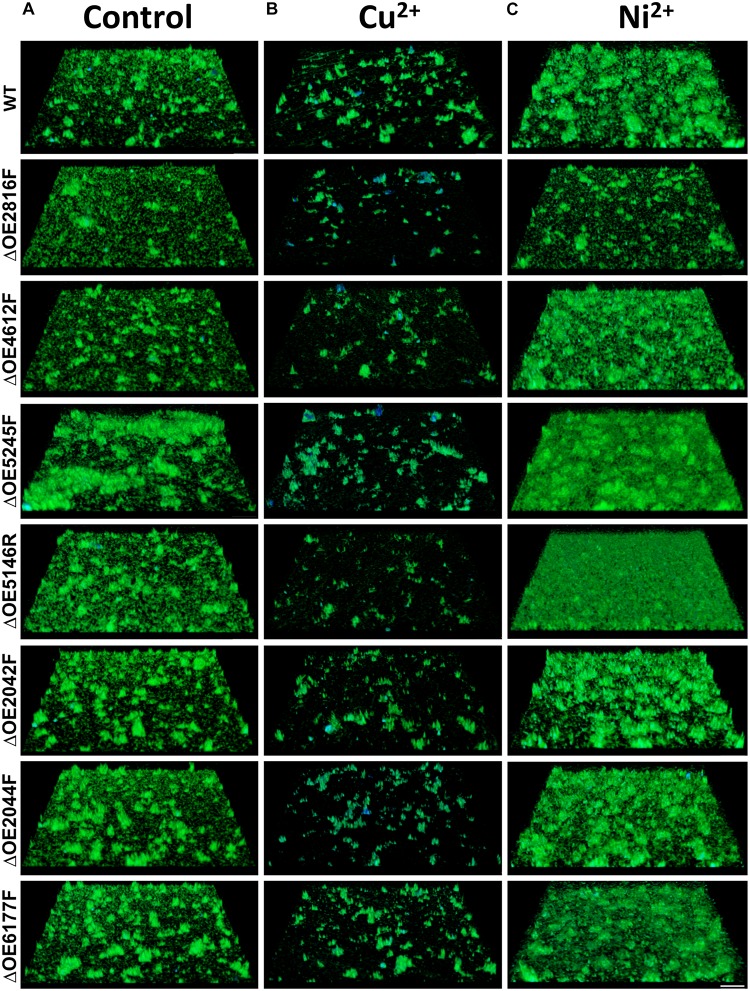
Confocal laser scanning micrographs (CLSM) of biofilms formed by *H. salinarum* wild type and deletion strains after exposure to metal ions. Biofilms were grown for 14 days in complex medium and were either incubated without treatment **(A)** or treated with 5 mM Cu^2 +^ **(B)** or 40 mM Ni^2 +^ **(C)** for 24 h. Genomic DNA was stained using acridine orange (green) and glycoconjugates (α-mannopyranosyl and α-glucopyranosyl residues) were stained with concanavalin A (ConA) Alexa Fluor^®^ (blue). Scale bar equals 25 μm. The surface coverage of these biofilms is quantified in Supplementary Figure S1.

Confocal laser scanning microscopy was used to assess the biofilm formation capabilities of the gene deletion mutants in comparison to the wild type strain R1 (Figure 8) and the surface coverage was quantified (Supplementary Figure S1). Biofilms were examined either untreated as well as after exposure to Cu^2+^ or Ni^2+^, respectively.

The strongest effect on biofilm formation was observed with OE5245F, indicating an increased biofilm formation and strongly altered architectures with extensive cellular aggregates in the untreated cells (Figure 8A). Deletions of OE2042F, OE2044F, OE5146R, and OE6177F, respectively, also increased biofilm formation and lead to higher numbers of macro-colonies with larger sizes compared to the parental strain.

Copper ion treatment of wild type biofilms leads to marked detachment of the cells from the surface and increased cellular aggregation compared to the untreated condition (Figure 8B), which is in accordance with the SEM analyses. Within the cellular aggregates, an increased amount of glycoconjugates (blue signal) was observed. Detachment and biofilm disintegration after Cu^2+^ exposure was even more pronounced in strains harboring the OE2816F, OE4612F, and OE5146R deletions, respectively.

Considerably increased biofilm formation and large macro-colonies were observed after Ni^2+^ treatment of wild type biofilms (Figure 8C), similar to the SEM analyses. Even more enhanced biofilm formation was observed with ΔOE5146R and ΔOE5245F in the presence of Ni^2+^. However, both strains differed with regard to their biofilm architectures. ΔOE5146R formed densely packed carpet-like biofilms with a smooth biofilm surface texture, whereas ΔOE5245F formed dense biofilms with pronounced protruding cellular aggregates, giving the biofilm surface a rough texture. In contrast, ΔOE2816F showed no alterations of the biofilm architecture after Ni^2+^ treatment, indicating an important role in the nickel-induced biofilm architecture (Figure 8 and Supplementary Figure S1). An overview of all these results is given in Table 3.

**TABLE 3:** Effects of gene deletion variants compared to *H. salinarum* R1 wild type^1^.

**Gene knockout strain**	**Planktonic growth**	**Adhesion**	**Metal ion sensitivity**		**Adhesion after metal ion treatment**		**Biofilm architecture after metal ion treatment^∗^**		
			**Cu^2+^**	**Ni^2+^**	**Cu^2+^**	**Ni^2+^**	**Control^2^**	**Cu^2+^**	**Ni^2+^**
ΔOE2816F	−	0	0	+	0	0	0	+ ↓	++↓
ΔOE4612F	0	0	0	+	0	0	0	+ ↓	0
ΔOE5245F	0	0	0	+ +	+	+	+ +↑	0	+ + ↑
ΔOE5146R	0	0	0	0	0	0	+ ↑	+↓	+↑
ΔOE2042F	+	+++	++	0	0	0	+ ↑	0	0
ΔOE2044F	+	+	0	0	0	0	+ ↑	0	0
ΔOE6177F	0	0	0	+	0	0	+ ↑	0	0

*^1^Effects of gene deletion mutants compared to the wild type on planktonic growth, adhesion, metal ion sensitivity, adhesion after metal ion treatment, and biofilm architecture after metal ion treatment were classified into four categories: 0 = no effect, + = moderate effect, ++ = strong effect, +++ = very strong effect. ^∗^↑ = increase in biofilm mass, ↓ = decrease in biofilm mass. ^2^Comparison of the biofilm architecture between gene deletion strains and the wild type without metal ion treatment.*

## Discussion

### SWATH Mass Spectrometry Identifies Adaptations in Response to Metal Stress

In the present study, a global proteome approach was applied to investigate the effects of copper and nickel ions on the proteins of planktonic and biofilm cells of *H. salinarum* R1. A total of 1378 proteins were detected corresponding to 54% proteome coverage. Quantitative data were obtained for 1180 proteins, i.e., 46% of the total proteins, across all samples. In a previous proteome study, investigating the general process of biofilm formation in this species, a 63% proteome coverage (corresponding to 1629 proteins) and a quantitation success rate of 57% was achieved (Losensky et al., 2017). The somewhat lower proteome coverage and quantitation success rate was possibly due to slightly differing protein isolation and purification procedures.

However, broad insights into the proteome changes in response to metal ion treatment were gained. These alterations included diverse functional categories, such as transcription or transport processes. At least 29% of the proteins showing significant abundance changes fall in the category of poorly characterized proteins. This underlines the limited knowledge with regard to metal resistance and utilization in *Halobacterium* and therefore necessitates further molecular biological investigations.

### Planktonic Cells Show Broader Proteome Changes Than Biofilms Cells After Metal Ion Treatment

Our proteome analysis identified distinct proteome responses in consequence of metal treatment in planktonic cells of *H. salinarum* strain R1. A total of 159 (6.2% of the proteome) and 186 (7.2%) of the quantified proteins showed significant changes after copper and nickel ion treatment, respectively. The major fraction, i.e., 111 of these proteins, was changed under both conditions, potentially constituting a general metal response. The remainder of these proteins possibly represents a metal-specific and adaptable subset of the proteome. Previously performed transcriptome studies with the closely related *H. salinarum* NRC-1 are based on planktonic cultures exposed to metal ions, since this strain is unable to form biofilms (Kaur et al., 2006). Metal ion exposure resulted in transcriptional changes for 2.9 and 3.0% of the total genes after nickel or copper ion treatment, respectively. Although the experimental setup was different and the applied metal ion concentrations were considerably lower compared to our study, similar conclusions were drawn from microarray data. The systems analysis that investigated effects of several other transition metals indicated the ability of *Halobacterium* to differentiate even between closely related metal ions to facilitate generalized as well as specific responses (Kaur et al., 2006).

Treatment of *H. salinarum* R1 biofilms went along with less profound but still distinct changes compared to the untreated controls. This suggests reduced susceptibility of biofilms to metal ion stress, which is in accordance with the previous observations that cells in biofilms of *H. salinarum* R1 show higher survival rates upon metal ion exposition (Völkel et al., 2018). A higher heavy metal resistance of biofilms compared with planktonic cells was also observed with several bacterial and eukaryotic biofilm forming organisms, such as *Pseudomonas aeruginosa* or *Candida albicans* (Teitzel and Parsek, 2003; Harrison et al., 2006).

Significant and strong changes of a small subset of the proteome occurred in biofilms of *H. salinarum* R1 as well, i.e., 29 and 20 proteins after copper or nickel ion treatment, respectively. These alterations are indicative of metal-specific responses in biofilms. Given the extensive rearrangements in biofilm structure after metal treatment, as observed by SEM and CLSM, the relatively low numbers of significantly altered proteins are rather unexpected. On the other hand, we observed severe effects on biofilm formation and architecture in consequence of individual gene deletions. The inactivation of certain selected genes from different functional categories resulted in diverse effects on growth, biofilm formation, and metal sensitization (see below) (Harrison et al., 2006), underlining that the metal responses are multifaceted in *H. salinarum*, similar to other microbes (Harrison et al., 2006; Cao et al., 2012).

### Extracellular Polymeric Substances in Biofilms Contribute to Metal Ion Resistance

Our proteome data showed distinct differences between planktonic cells and biofilms treated with metal ions. While metal ion treatment of planktonic cells lead to significant changes of the proteome, biofilms showed less and mostly metal-specific effects. Considering the metal ion concentrations in treated planktonic and biofilm cells, as measured by AAS, the planktonic cells were exposed to 9- to 35-fold higher levels of Ni^2+^ and Cu^2+^ than biofilm cells. The higher concentrations obviously lead to stronger responses, respectively, proteome changes in the planktonic cells, that are mostly independent of the metal ion present. Regarding the biofilm samples, 65–83% of the metal ions were detected in EPS-fractions, implying that EPS serves as a sink and binds most of the metal ions. The biosorption of metal ions by EPS was also shown in haloalkaliphilic *Bacillus* species with a maximum of 90% binding of lead (Shameer, 2016), or a 75% sequestration of Cu^2+^ in polysaccharides produced by *Bacillus firmus* (Salehizadeh and Shojaosadati, 2003).

Our study showed that the EPS in *H. salinarum* is dominated by proteins, i.e., 52 mg/g dry mass, while the amount of carbohydrates and uronic acids (16 and 6 mg/dry mass, respectively) is rather low. This is in contrast to studies on EPS in bacterial and other archaeal species, where EPSs are mainly dominated by polysaccharides (Rode, 2004; Jachlewski et al., 2015). Since *H. salinarum* is not capable of sugar degradation, polysaccharides apparently play no important role in extracellular nutrient supply and therefore might only be present in small amounts (Rawal et al., 1988; Falb et al., 2008). Previous fluorescence microscopic studies on haloarchaeal biofilms indicated the presence of extracellular DNA (Fröls et al., 2012). Using an SYBR Green-based method (Leggate et al., 2006), the eDNA amounts in biofilms in the present study were not quantifiable. eDNA can play structural roles and interact by electrostatic forces (Heijstra et al., 2009), but most likely under low salt conditions. Recent studies on the EPS of different microbial communities have shown that the number of electrostatic binding sites is about 20- to 30-fold higher in EPS compared to the bacterial cell surface (Liu and Fang, 2002). Consequently, the EPS matrix serves as a diffusion barrier, preventing or at least decreasing the permeation of charged heavy metal ions (Harrison et al., 2007).

Metal ion treatment of *H. salinarum* mature biofilms affected the protein content in EPS with a 0.6 fold decrease in Cu^2+^ treated and a 2.7-fold increase in Ni^2+^ treated biofilms. Higher EPS amounts, i.e., total carbohydrate content, upon increased metal concentrations were observed in *Pantoea agglomerans* (Mohite et al., 2018) and *Pseudoalteromonas* sp. that enhance the production of EPS as a result of increasing mercury and cadmium ion concentrations (Caruso et al., 2018). The authors hypothesize that the increased tolerance against metal ions depends of the presence of uronic acids and sulfate groups capable of binding cations in EPS. In our study, the exposure to nickel ions resulted in an increased ratio of EPS mass compared to total biofilm mass, i.e., 2.5-fold increase in the amount of proteins. The finding suggests a role of the potentially versatile functional groups of proteins in metal ion binding in the EPS of *H. salinarum.* Taken together our data suggested extensive biosorption of metal ions within the EPS that probably contributes to the increased metal ion resistances observed in *H. salinarum* biofilms exposed to Cu^2+^ or Ni^2+^ (Völkel et al., 2018).

### Gene Deletions in *H. salinarum* R1 Resulted in Diverse Effects in Response to Metal Ion Stress

Besides EPS, it is reasonable to assume that further resistance mechanisms exist, as suggested by the proteome changes observed here. A potential role of individual genes from different functional categories was tested by gene deletion studies.

A serine protease (protein ID: B0R859) and an uncharacterized protein (protein ID: B0R587) showed the highest abundance in Ni^2+^-exposed biofilms, suggesting a specificity for nickel ions. The deletion of the respective genes OE4612F and OE2816F resulted in an increased Ni^2+^ sensitivity and in case of OE2816F in significant differences of the biofilm architecture after Ni^2+^ exposure. Both proteins contain a twin arginine transport (TAT) sequence, indicating they are exported and are located close to the membrane or outside the cell. B0R587 was found in the membrane proteome of *H. salinarum* R1 (Bisle, 2006), but the function of this protein is still unknown. However, the significant changes of the biofilm architecture in gene deletion mutants exposed to Ni^2+^ suggest an important role of B0R587 in biofilm rearrangement and possibly in EPS production. The serine protease halolysin (B0R859) belongs to the extracellular proteases, enabling the degradation of proteins and peptides in the environment to generate oligonucleotides, dipeptides, and amino acid intermediates for uptake and fueling the central cell metabolism (De Castro et al., 2006). The increased abundance of this protease in Ni^2+^-exposed biofilms may increase the amount of negatively charged binding sites embedded in EPS and thus enable an improved protection of cells in biofilm.

The P-type transport ATPase CopA (B0R3Z0) was increased in Cu^2+^ exposed planktonic cells in this study. An increase in the expression of the corresponding gene *copA/yvgX* in response to Cu^2+^ treatment was also observed in recent transcription studies with *H. salinarum* (Kaur et al., 2006; Völkel et al., 2018). The deletion of *copA* resulted in a significantly increased sensitivity to Cu^2+^, whereas no effects were observed when the cells were treated with Ni^2+^. Also in *H. salinarum* NRC-1, the deletion of *yvgX* resulted in a defective growth only in the presence of copper ions, suggesting a Cu^2+^-specific efflux ATPase (Kaur et al., 2006). In contrast, the deletion of the HMA-domain protein (B0R3Z1), a putative Cu^2+^ trafficking chaperone associated with CopA, showed no effects after Cu^2+^ or Ni^2+^ treatment. Thus, additional Cu^2+^ binding proteins might be involved in the CopA-mediated Cu^2+^ export.

Besides P-type ATPases, the large protein family of ATP-binding cassette (ABC) transporters may also contribute to metal resistance in haloarchaea. Among the ABC transporter subunits detected in our study, about 80% were decreased after metal ion treatment in planktonic cultures. In biofilms, significantly less ABC transporter proteins were affected by metal treatment with decreased amounts of two proteins in the case of Cu^2+^ treatment and an increase of only one ABC transporter ATP binding protein (B0R9Z2) in Ni^2+^ treated biofilms. The deletion of the B0R9Z2 encoding gene resulted in a higher sensitivity to nickel ions, and a significantly denser biofilm architecture in both, Ni^2+^ treated and untreated control biofilms. The ATP-binding protein ZnuC, a component of the ZnuABC transporter, was increased only in planktonic cultures exposed to Ni^2+^. The deletion of *znuC* also resulted in denser biofilms in the case of Ni^2+^-exposed samples and untreated controls, suggesting a role of these proteins in the transport of Ni^2+^. ABC transporters are involved in a variety of physiological functions, like nutrient uptake, transport of proteins, or drug efflux. Besides the transport of specific substrates, some ABC transporters have multiple specificities, enabling the transport of metal ions (Salt and Wagner, 1993; Srivastava and Kowshik, 2013). The significant decrease of ABC transporter proteins in planktonic cells exposed to metal ions may serve as a resistance mechanism to reduce an import of metal ions via non-specific transporters. However, deletion of a single ABC transporter gene possibly has no severe effects on the microorganisms, since the large repertoire of ABC transporters could compensate this effect (Srivastava and Kowshik, 2013).

Resistance mechanisms in response to environmental stress are mainly based on global transcriptional changes. In the proteome study presented here, about 75% of the significantly changed proteins in the category “information storage and processing” were 2- to 15-fold increased in planktonic cells in the case of Cu^2+^ or Ni^2+^ treatment, respectively, suggesting transcriptional regulations to overcome the metal stress. Microarray analysis of the closely related *H. salinarum* NRC-1 also showed transcriptional changes in response to metal ions in planktonic cells. Among the genes affected, 69% showed significantly changed mRNA levels within 0–25 min after metal exposure (Kaur et al., 2006). Besides changes of Lrp/AsnC-, Cro/C1-, or ArsR family transcription regulators, the amounts of several PadR family transcription regulators were prominently altered in our study. As an example, the PadR family regulator B0R8N0 was increased in both, planktonic and in biofilm cells after Cu^2+^ or Ni^+^ treatment. However, deletion of the respective gene did not result in significant effects when the cells were treated with these metal ions. Due to the large repertoire of nine PadR family transcription regulators in *H. salinarum*, the function of one deleted regulator might be complemented by the others.

## Conclusion

Our study demonstrated the diverse effects of heavy metal ions on cell growth and biofilm architecture in *H. salinarum* R1. Investigations of the EPS showed that most of the metals in biofilms are absorbed, indicating an important role of the EPS in metal resistance of cells living as biofilm. Our proteomic approach identified proteins associated with metal stress and biofilm rearrangement in *H. salinarum* R1 after Cu^2+^ or Ni^2+^ treatment. Each of the targets selected for the subsequent gene deletion studies showed metal-induced effects with regard to at least one of the characteristics under investigation, i.e., growth, adhesion, or biofilm formation, respectively. However, future studies are required to unravel their exact cellular roles and interplay.

## Data Availability Statement

The mass spectrometry proteomics data have been deposited to the ProteomeXchange Consortium via the PRIDE (Perez-Riverol et al., 2019) partner repository with the dataset identifier PXD015192.

## Author Contributions

SV, FP, and GL planned the study. NB performed the SEM analysis. CL conducted the mass spectrometry analysis and data processing. SH executed the statistical analysis. SV carried out all other analysis. All authors discussed the results, wrote the manuscript, and approved the final version of the manuscript.

## Conflict of Interest

The authors declare that the research was conducted in the absence of any commercial or financial relationships that could be construed as a potential conflict of interest.
