# Supplementary material for: How to Cope With Heavy Metal Ions: Cellular and Proteome-Level Stress Response to Divalent Copper and Nickel in Halobacterium salinarum R1 Planktonic and Biofilm Cells
Source: Front Microbiol. 2020 Jan 17;10:3056. doi: 10.3389/fmicb.2019.03056 (PMC6978704; doi:10.3389/fmicb.2019.03056)
Supplement: Supplementary file 1 [file Table_1.DOCX]

**How to Cope with Heavy Metal Ions: Cellular and Proteome-Level Stress Response to Divalent Copper and Nickel in *Halobacterium salinarum* R1 Planktonic and Biofilm Cells**

**Sabrina Völkel, Sascha Hein, Nathalie Benker, Felicitas Pfeifer, Christof Lenz, Gerald Losensky***

***Correspondence:**Dr. Gerald Losensky
E-mail: losensky@bio.tu-darmstadt.de

# Supplementary Material

Table S1: Oligonucleotides used in this study.

| Name | Oligonucleotide sequence (5’-3’) |
| --- | --- |
| Construction of deletion mutants | |
| US-OE4612F-FWD | tgaccatgggatcgtgatctcgtggtgcacc |
| US-OE4612F-REV | cgtccggtcgacccactcgccaggacagtgtac |
| DS-OE4612F-FWD | tggcgagtgggtcgaccggacgcgattctgactg |
| DS-OE4612F-REV | agtaagcttggacccgagcagggtcttgccg |
| US-OE5245F-FWD | agtccatggcgtcgaaatggtcgaaacgacc |
| US-OE5245F-REV | tgtggtcgccgagaccccacggtcggttcgtaa |
| DS- OE5245F-FWD | ccgtggggtctcggcgaccacaccgtagttagt |
| DS-OE5245F-REV | tgaaagcttcccactccacacgcttgttccg |
| US-OE5146R-FWD | agtctgcagaacctcaccgaggggttcgttc |
| US- OE5146R-REV | cacgtccgtcatcgaagttcctccatgtggcgttc |
| DS- OE5146R-FWD | aggaacttcgatgacggacgtgatcgtccagcc |
| DS- OE5146R-REV | tgatctagaatggcggcgctgtcggtggaga |
| US-OE2042F-FWD | agtccatggcgtgctcacgcagtcgatgacg |
| US-OE2042F-REV | ccacaccccggcactgaagactacggcatcccg |
| DS-OE2042F-FWD | agtcttcagtgccggggtgtggcggcgcgaa |
| DS-OE2042F-REV | tgatctagagctgtgagagccgggagttctc |
| US-OE2044F-FWD | agtccatggcgacaacgaacgcaccgcgcg |
| US-OE2044F-REV | gactggcggccgcgaccggcgctactcgatcaa |
| DS-OE2044F-FWD | agcgccggtcgcggccgccagtcaccgtttgtgc |
| DS-OE2044F-REV | tgatctagacgcactcaccgaactcggtcag |
| US-OE6177F-FWD | agtctgcagccaacccacgtcaggatctattaa |
| US-OE6177F-REV | aaaccgggaagccgtctctttgccttctactgg |
| DS-OE6177F-FWD | caaagagacggcttcccggttttcagcgatcag |
| DS-OE6177F-REV | tgatctagatggggtcgtctaccgcttgcag |
| US-OE2816F-FWD | accgtgagagacactagtggatcccccgggctgcaaacggtctatagcgtccccgagccg |
| US-OE2816F-REV | actgcgccaaaccacccgtgcgttgatttatgtttcaactaaaatatgtgctgtggttcg |
| DS-OE2816F-FWD | gaaacataaatcaacgcacgggtggtttggcgcagtgtgccag |
| DS-OE2816F-REV | gccactcttcacacgcggtacctctagaatcgatagctagtttacaatagtagtgagattgaacaatcttcgtttaaagtttacgttgctaaaggggatatagtcc |
| Screening of deletion mutants | |
| OE4612F-FWD | ctcgaacgcagcgtggctgtgaccg |
| OE4612F-REV | cacggagaggcggcgcaggagc |
| OE5245F-FWD | cgagatcgaacagatgatcgtcg |
| OE5245F-REV | cgtcccactccacacgcttgtt |
| OE5146R-FWD | tgaccgcgcaatcgaatcactgg |
| OE5146R-REV | cgttgaaactgcgagcgacctgt |
| OE2042F-FWD | gatctggctggtgaacacgtgca |
| OE2042F-REV | ctcgatccggtcgacgaagaagc |
| OE2044F-FWD | gcagtcgatgccttcgagaacgt |
| OE2044F-REV | cgtcgttggtcgggaagaaatggc |
| OE6177F-FWD | actgcgcagatgaccatcaggag |
| OE6177F-REV | cgcgagagctgattatccgcaac |
| OE2816F-FWD | cggcctccacgacgacttagac |
| OE2816F-REV | cggacccgattccgatcgtaatg |
