# Supplementary material for: How to Cope With Heavy Metal Ions: Cellular and Proteome-Level Stress Response to Divalent Copper and Nickel in Halobacterium salinarum R1 Planktonic and Biofilm Cells
Source: Front Microbiol. 2020 Jan 17;10:3056. doi: 10.3389/fmicb.2019.03056 (PMC6978704; doi:10.3389/fmicb.2019.03056)
Supplement: Supplementary file 4 [file Image_1.pdf]

How to Cope with Heavy Metal Ions: Cellular and Proteome-Level Stress Response to Divalent Copper and Nickel in *Halobacterium salinarum* R1 Planktonic and Biofilm Cells

Sabrina Völkel, Sascha Hein, Nathalie Benker, Felicitas Pfeifer, Christof Lenz, Gerald Losensky*

***Correspondence:**
Dr. Gerald Losensky
E-mail: losensky@bio.tu-darmstadt.de

1 Supplementary Figures

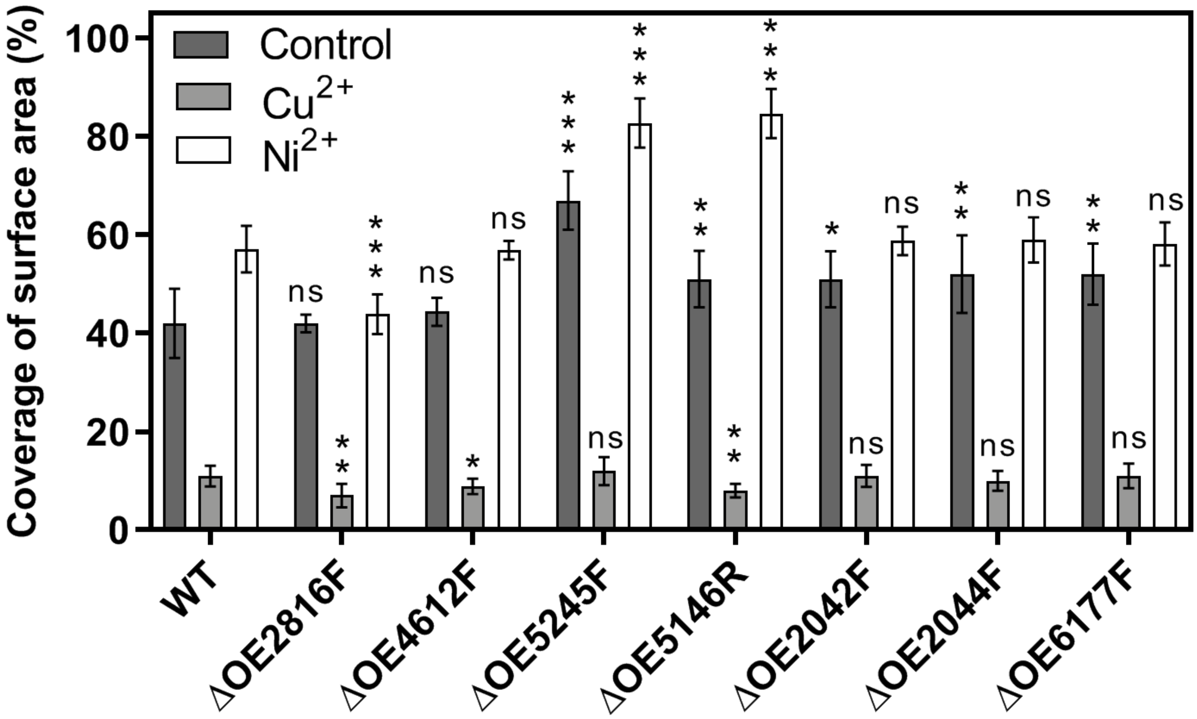

Figure S1. Quantification of surface coverage in biofilms formed by *H. salinarum* wild type and deletions strains after exposure to metal ions. The quantification of the surface coverage was based on confocal laser scanning micrographs of biofilms exposed to copper- and nickel ions as well as untreated biofilms (control). For each condition and strain at least eight micrographs were analyzed. The significance of the surface coverage in deletion strains compared to the wild type strain was determined by *t*-test (ns, not significant, * significant = $p < 0.05$, ** highly significant = $p < 0.01$, *** extremely significant = $p < 0.001$).
